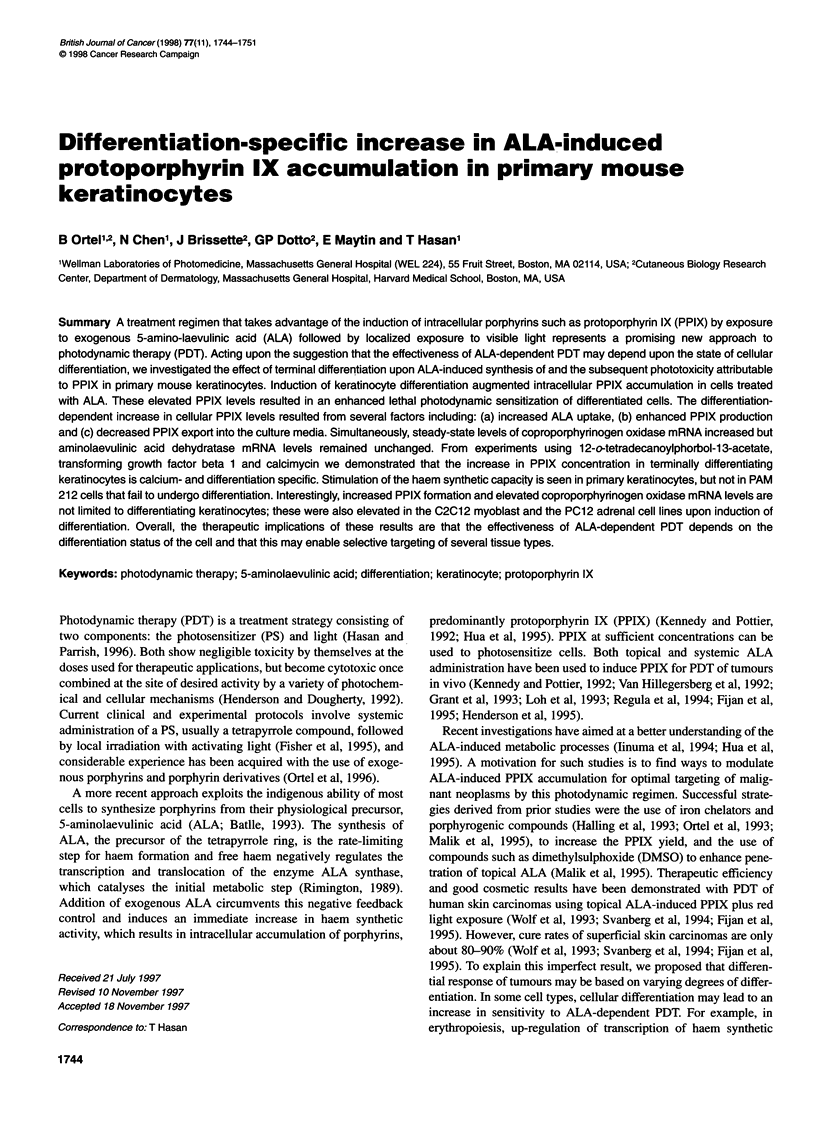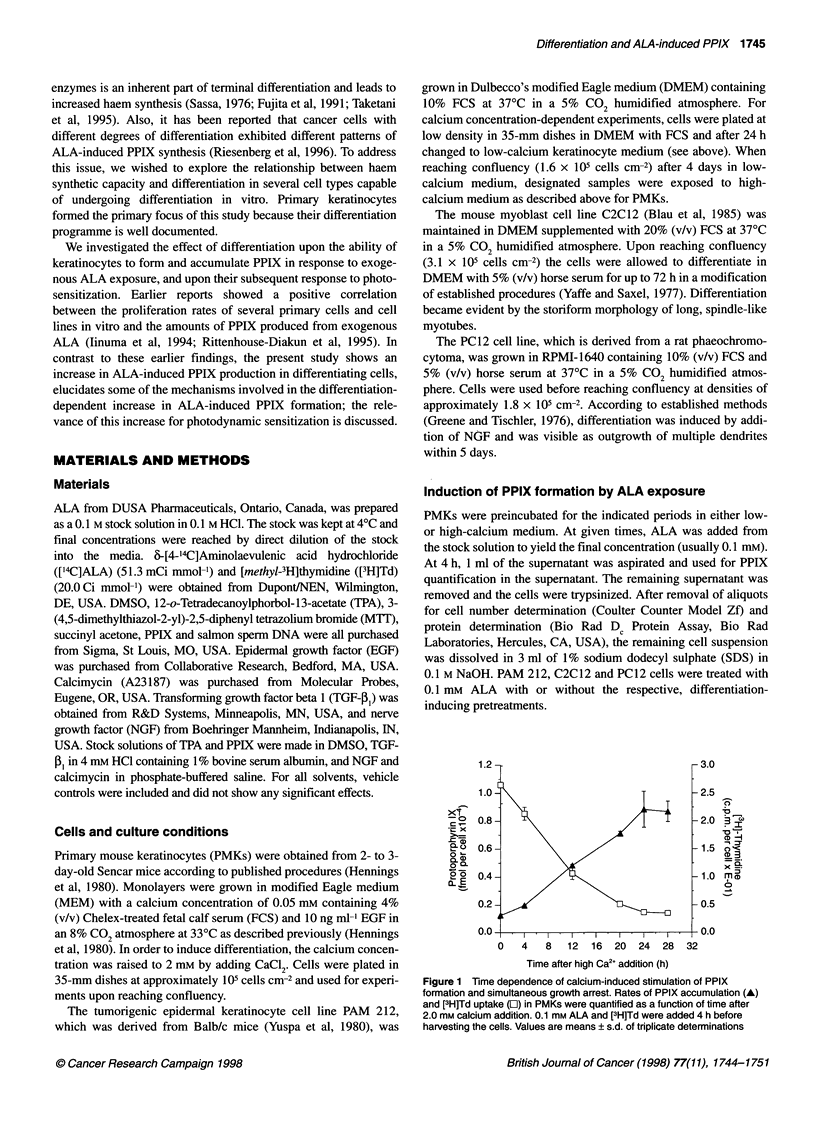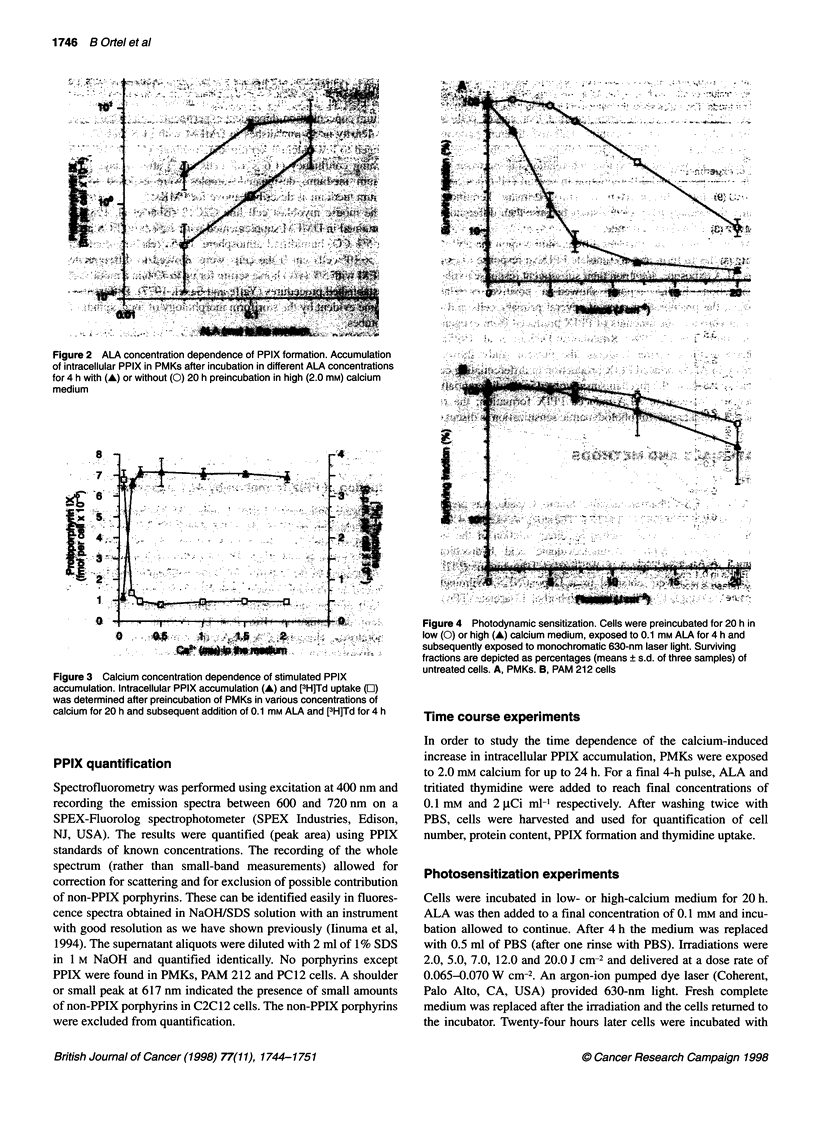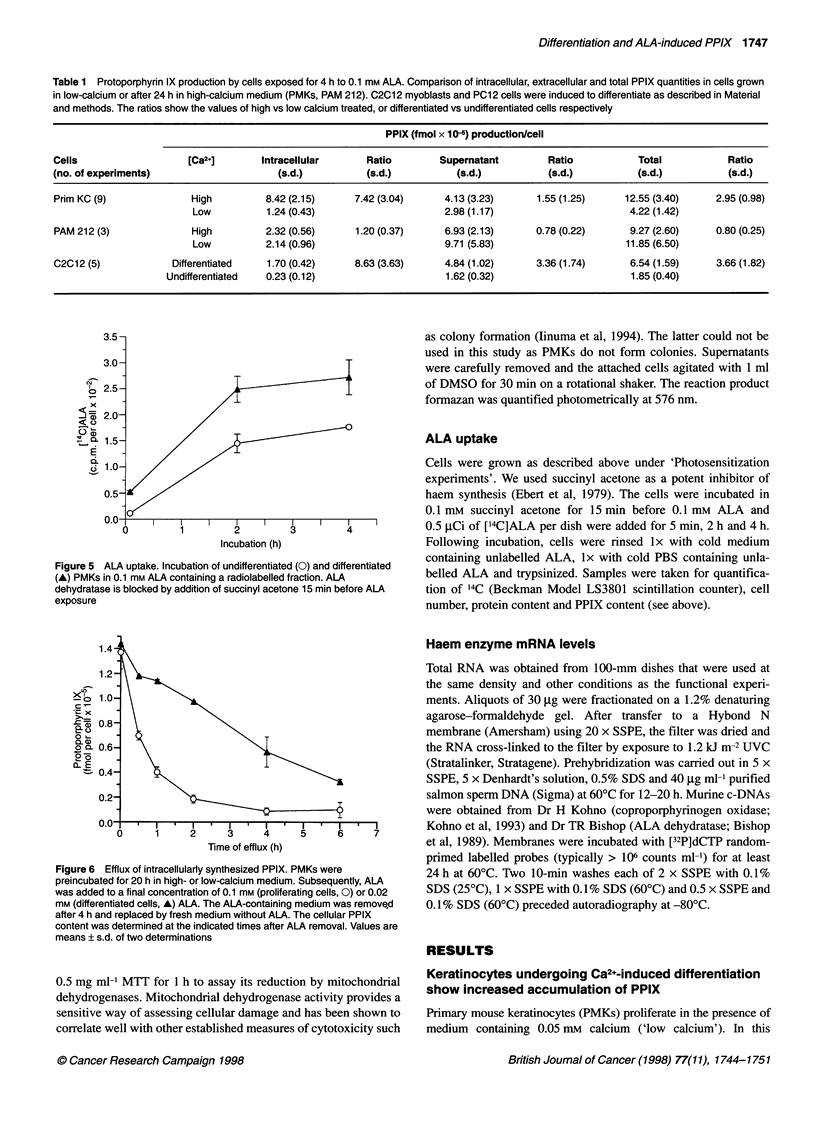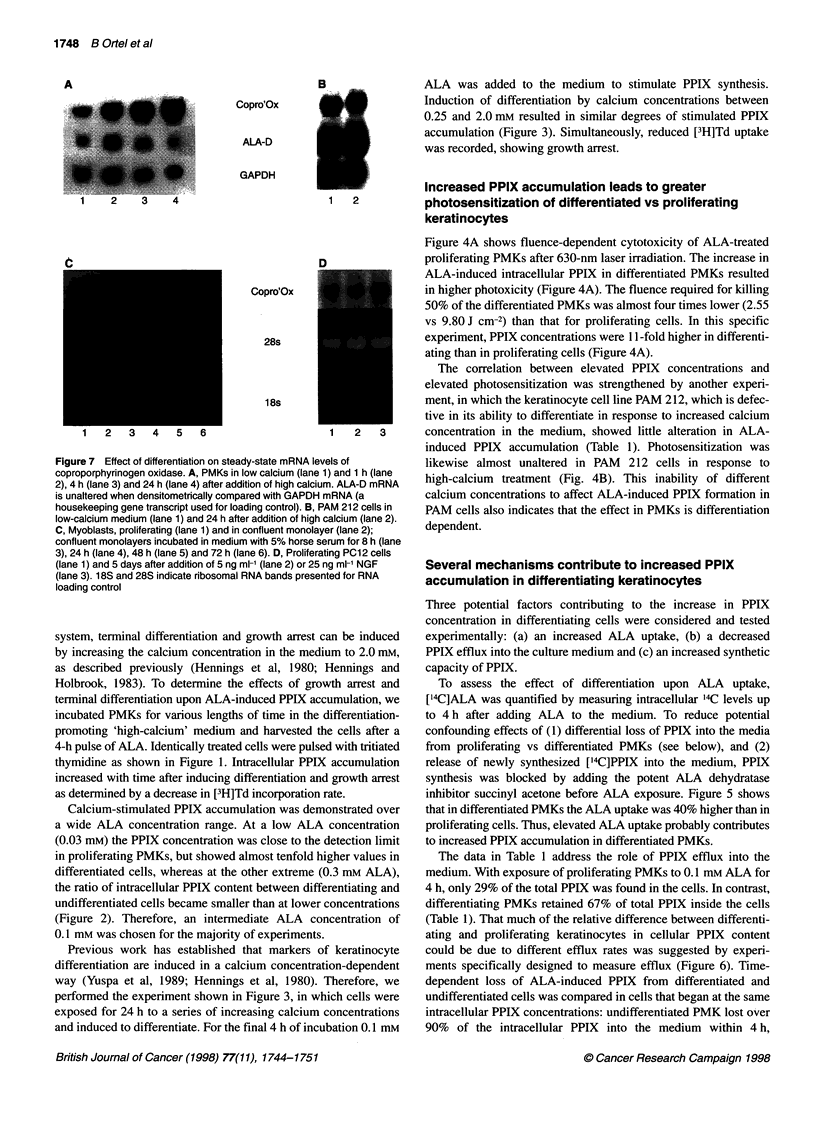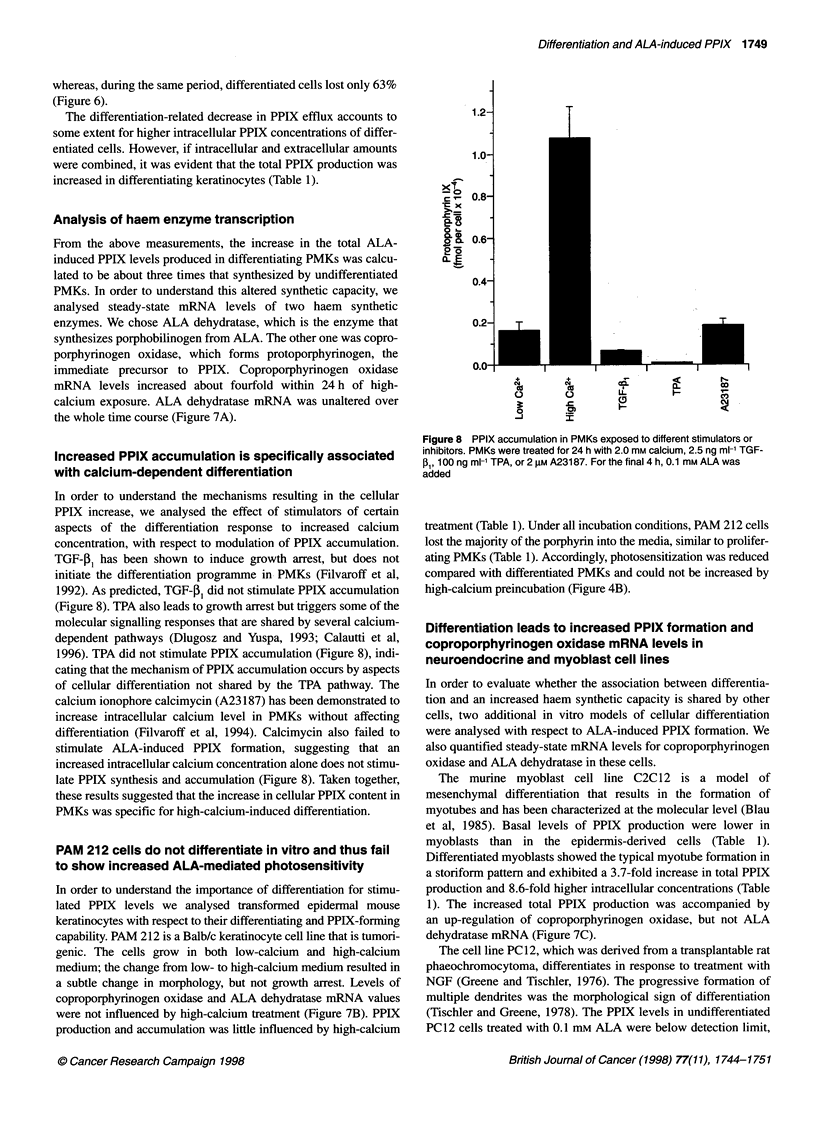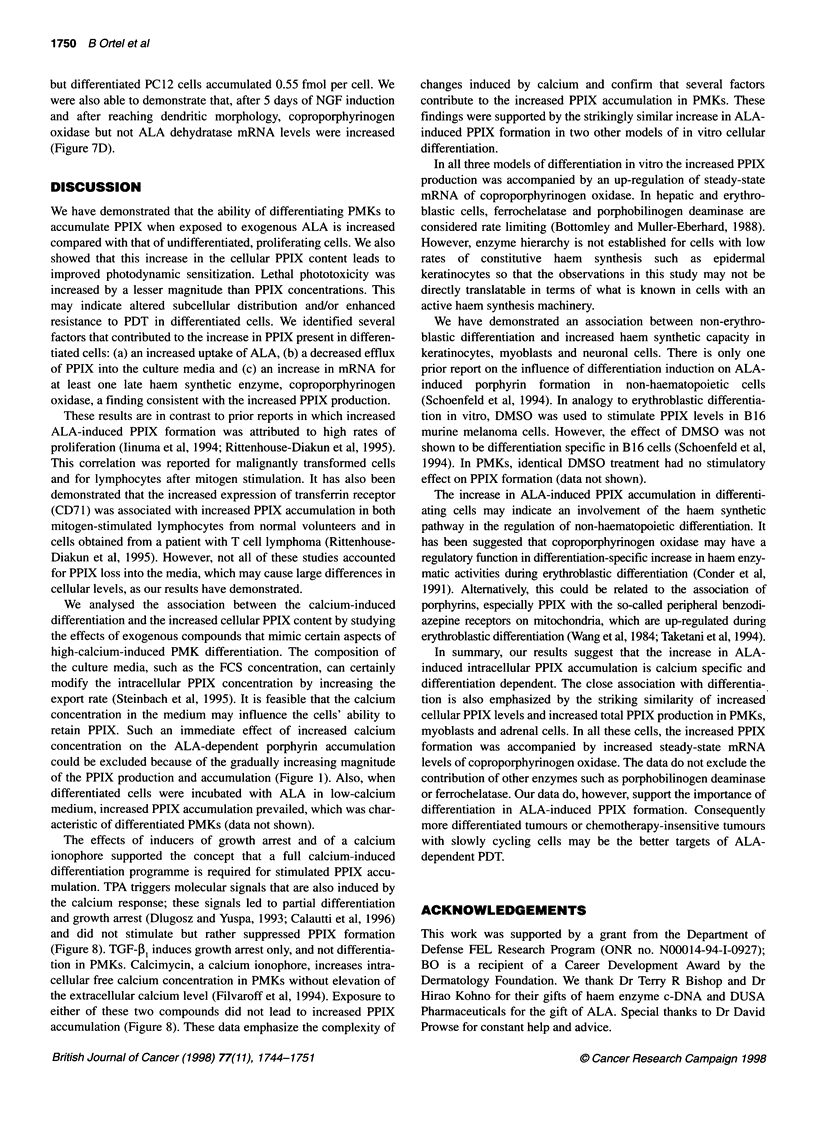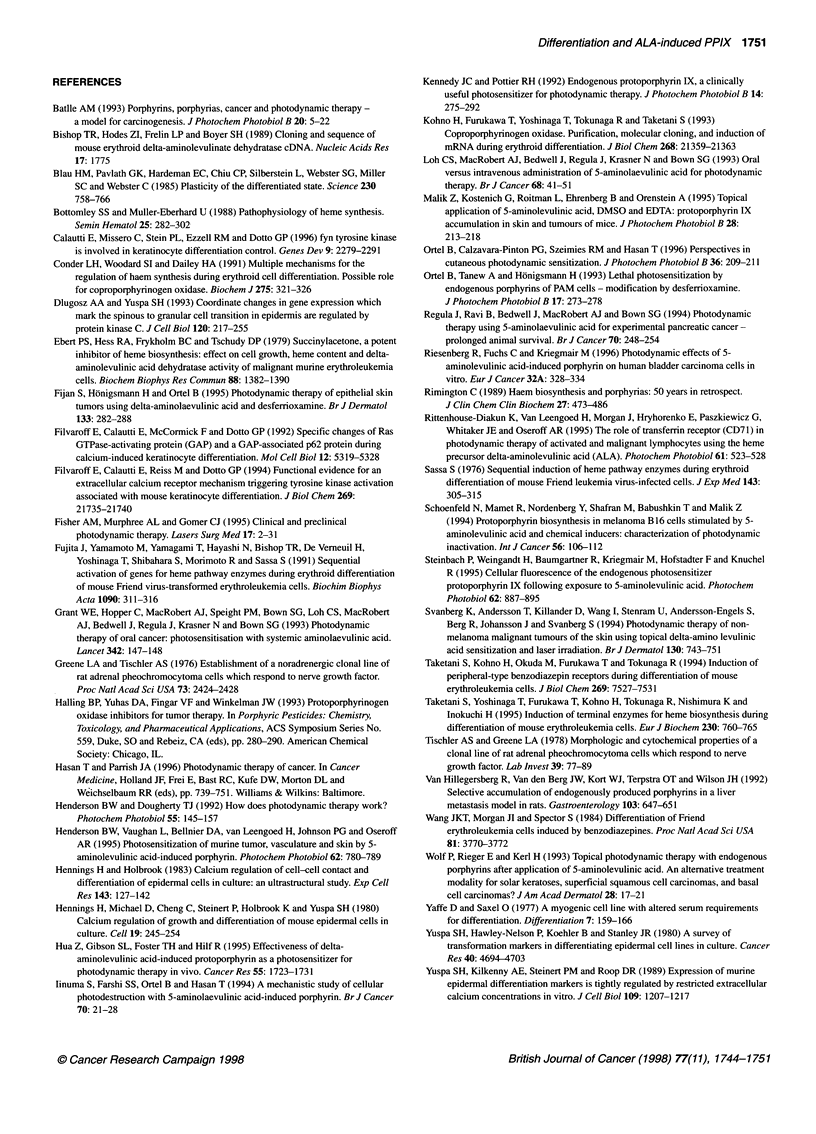# Differentiation-specific increase in ALA-induced protoporphyrin IX accumulation in primary mouse keratinocytes.

**DOI:** 10.1038/bjc.1998.292

**Published:** 1998-06

**Authors:** B. Ortel, N. Chen, J. Brissette, G. P. Dotto, E. Maytin, T. Hasan

**Affiliations:** Wellman Laboratories of Photomedicine, Massachusetts General Hospital, Boston 02114, USA.

## Abstract

**Images:**


British Journal of Cancer (1998) 77(11), 1744–1751
© 1998 Cancer Research Campaign

### Differentiation-specific increase in ALA-induced protoporphyrin IX accumulation in primary mouse keratinocytes

**B Ortel[1,2], N Chen[1], J Brissette[2], GP Dotto[2], E Maytin and T Hasan[1]**

[1]Wellman Laboratories of Photomedicine, Massachusetts General Hospital (WEL 224), 55 Fruit Street, Boston, MA 02114, USA; [2]Cutaneous Biology Research Center, Department of Dermatology, Massachusetts General Hospital, Harvard Medical School, Boston, MA, USA

**Summary** A treatment regimen that takes advantage of the induction of intracellular porphyrins such as protoporphyrin IX (PPIX) by exposure to exogenous 5-amino-laevulinic acid (ALA) followed by localized exposure to visible light represents a promising new approach to photodynamic therapy (PDT). Acting upon the suggestion that the effectiveness of ALA-dependent PDT may depend upon the state of cellular differentiation, we investigated the effect of terminal differentiation upon ALA-induced synthesis of and the subsequent phototoxicity attributable to PPIX in primary mouse keratinocytes. Induction of keratinocyte differentiation augmented intracellular PPIX accumulation in cells treated with ALA. These elevated PPIX levels resulted in an enhanced lethal photodynamic sensitization of differentiated cells. The differentiation-dependent increase in cellular PPIX levels resulted from several factors including: (a) increased ALA uptake, (b) enhanced PPIX production and (c) decreased PPIX export into the culture media. Simultaneously, steady-state levels of coproporphyrinogen oxidase mRNA increased but aminolaevulinic acid dehydratase mRNA levels remained unchanged. From experiments using 12-*o*-tetradecanoylphorbol-13-acetate, transforming growth factor beta 1 and calcimycin we demonstrated that the increase in PPIX concentration in terminally differentiating keratinocytes is calcium- and differentiation specific. Stimulation of the haem synthetic capacity is seen in primary keratinocytes, but not in PAM 212 cells that fail to undergo differentiation. Interestingly, increased PPIX formation and elevated coproporphyrinogen oxidase mRNA levels are not limited to differentiating keratinocytes; these were also elevated in the C2C12 myoblast and the PC12 adrenal cell lines upon induction of differentiation. Overall, the therapeutic implications of these results are that the effectiveness of ALA-dependent PDT depends on the differentiation status of the cell and that this may enable selective targeting of several tissue types.

**Keywords:** photodynamic therapy; 5-aminolaevulinic acid; differentiation; keratinocyte; protoporphyrin IX

Photodynamic therapy (PDT) is a treatment strategy consisting of two components: the photosensitizer (PS) and light (Hasan and Parrish, 1996). Both show negligible toxicity by themselves at the doses used for therapeutic applications, but become cytotoxic once combined at the site of desired activity by a variety of photochemical and cellular mechanisms (Henderson and Dougherty, 1992). Current clinical and experimental protocols involve systemic administration of a PS, usually a tetrapyrrole compound, followed by local irradiation with activating light (Fisher et al, 1995), and considerable experience has been acquired with the use of exogenous porphyrins and porphyrin derivatives (Ortel et al, 1996).

A more recent approach exploits the indigenous ability of most cells to synthesize porphyrins from their physiological precursor, 5-aminolaevulinic acid (ALA; Batlle, 1993). The synthesis of ALA, the precursor of the tetrapyrrole ring, is the rate-limiting step for haem formation and free haem negatively regulates the transcription and translocation of the enzyme ALA synthase, which catalyses the initial metabolic step (Rimington, 1989). Addition of exogenous ALA circumvents this negative feedback control and induces an immediate increase in haem synthetic activity, which results in intracellular accumulation of porphyrins, predominantly protoporphyrin IX (PPIX) (Kennedy and Pottier, 1992; Hua et al, 1995). PPIX at sufficient concentrations can be used to photosensitize cells. Both topical and systemic ALA administration have been used to induce PPIX for PDT of tumours in vivo (Kennedy and Pottier, 1992; Van Hillegersberg et al, 1992; Grant et al, 1993; Loh et al, 1993; Regula et al, 1994; Fijan et al, 1995; Henderson et al, 1995).

Recent investigations have aimed at a better understanding of the ALA-induced metabolic processes (Iinuma et al, 1994; Hua et al, 1995). A motivation for such studies is to find ways to modulate ALA-induced PPIX accumulation for optimal targeting of malignant neoplasms by this photodynamic regimen. Successful strategies derived from prior studies were the use of iron chelators and porphyrogenic compounds (Halling et al, 1993; Ortel et al, 1993; Malik et al, 1995), to increase the PPIX yield, and the use of compounds such as dimethylsulphoxide (DMSO) to enhance penetration of topical ALA (Malik et al, 1995). Therapeutic efficiency and good cosmetic results have been demonstrated with PDT of human skin carcinomas using topical ALA-induced PPIX plus red light exposure (Wolf et al, 1993; Svanberg et al, 1994; Fijan et al, 1995). However, cure rates of superficial skin carcinomas are only about 80–90% (Wolf et al, 1993; Svanberg et al, 1994; Fijan et al, 1995). To explain this imperfect result, we proposed that differential response of tumours may be based on varying degrees of differentiation. In some cell types, cellular differentiation may lead to an increase in sensitivity to ALA-dependent PDT. For example, in erythropoiesis, up-regulation of transcription of haem synthetic

*Received 21 July 1997*
*Revised 10 November 1997*
*Accepted 18 November 1997*

*Correspondence to:* T Hasan enzymes is an inherent part of terminal differentiation and leads to increased haem synthesis (Sassa, 1976; Fujita et al, 1991; Taketani et al, 1995). Also, it has been reported that cancer cells with different degrees of differentiation exhibited different patterns of ALA-induced PPIX synthesis (Riesenberg et al, 1996). To address this issue, we wished to explore the relationship between haem synthetic capacity and differentiation in several cell types capable of undergoing differentiation in vitro. Primary keratinocytes formed the primary focus of this study because their differentiation programme is well documented.

We investigated the effect of differentiation upon the ability of keratinocytes to form and accumulate PPIX in response to exogenous ALA exposure, and upon their subsequent response to photosensitization. Earlier reports showed a positive correlation between the proliferation rates of several primary cells and cell lines in vitro and the amounts of PPIX produced from exogenous ALA (Iinuma et al, 1994; Rittenhouse-Diakun et al, 1995). In contrast to these earlier findings, the present study shows an increase in ALA-induced PPIX production in differentiating cells, elucidates some of the mechanisms involved in the differentiation-dependent increase in ALA-induced PPIX formation; the relevance of this increase for photodynamic sensitization is discussed.

### MATERIALS AND METHODS

### Materials

ALA from DUSA Pharmaceuticals, Ontario, Canada, was prepared as a 0.1 M stock solution in 0.1 M HCl. The stock was kept at 4°C and final concentrations were reached by direct dilution of the stock into the media. δ-[4-$^{14}$C]Aminolaevulenic acid hydrochloride ([$^{14}$C]ALA) (51.3 mCi mmol$^{-1}$) and [*methyl*-$^{3}$H]thymidine ([$^{3}$H]Td) (20.0 Ci mmol$^{-1}$) were obtained from Dupont/NEN, Wilmington, DE, USA. DMSO, 12-*o*-Tetradecanoylphorbol-13-acetate (TPA), 3-(4,5-dimethylthiazol-2-yl)-2,5-diphenyl tetrazolium bromide (MTT), succinyl acetone, PPIX and salmon sperm DNA were all purchased from Sigma, St Louis, MO, USA. Epidermal growth factor (EGF) was purchased from Collaborative Research, Bedford, MA, USA. Calcimycin (A23187) was purchased from Molecular Probes, Eugene, OR, USA. Transforming growth factor beta 1 (TGF-$\beta_1$) was obtained from R&D Systems, Minneapolis, MN, USA, and nerve growth factor (NGF) from Boehringer Mannheim, Indianapolis, IN, USA. Stock solutions of TPA and PPIX were made in DMSO, TGF-$\beta_1$ in 4 mM HCl containing 1% bovine serum albumin, and NGF and calcimycin in phosphate-buffered saline. For all solvents, vehicle controls were included and did not show any significant effects.

### Cells and culture conditions

Primary mouse keratinocytes (PMKs) were obtained from 2- to 3-day-old Sencar mice according to published procedures (Hennings et al, 1980). Monolayers were grown in modified Eagle medium (MEM) with a calcium concentration of 0.05 mM containing 4% (v/v) Chelex-treated fetal calf serum (FCS) and 10 ng ml$^{-1}$ EGF in an 8% $CO_2$ atmosphere at 33°C as described previously (Hennings et al, 1980). In order to induce differentiation, the calcium concentration was raised to 2 mM by adding $CaCl_2$. Cells were plated in 35-mm dishes at approximately $10^5$ cells cm$^{-2}$ and used for experiments upon reaching confluency.

The tumorigenic epidermal keratinocyte cell line PAM 212, which was derived from Balb/c mice (Yuspa et al, 1980), was grown in Dulbecco's modified Eagle medium (DMEM) containing 10% FCS at 37°C in a 5% $CO_2$ humidified atmosphere. For calcium concentration-dependent experiments, cells were plated at low density in 35-mm dishes in DMEM with FCS and after 24 h changed to low-calcium keratinocyte medium (see above). When reaching confluency ($1.6 \times 10^5$ cells cm$^{-2}$) after 4 days in low-calcium medium, designated samples were exposed to high-calcium medium as described above for PMKs.

The mouse myoblast cell line C2C12 (Blau et al, 1985) was maintained in DMEM supplemented with 20% (v/v) FCS at 37°C in a 5% $CO_2$ humidified atmosphere. Upon reaching confluency ($3.1 \times 10^5$ cells cm$^{-2}$) the cells were allowed to differentiate in DMEM with 5% (v/v) horse serum for up to 72 h in a modification of established procedures (Yaffe and Saxel, 1977). Differentiation became evident by the storiform morphology of long, spindle-like myotubes.

The PC12 cell line, which is derived from a rat phaeochromocytoma, was grown in RPMI-1640 containing 10% (v/v) FCS and 5% (v/v) horse serum at 37°C in a 5% $CO_2$ humidified atmosphere. Cells were used before reaching confluency at densities of approximately $1.8 \times 10^5$ cm$^{-2}$. According to established methods (Greene and Tischler, 1976), differentiation was induced by addition of NGF and was visible as outgrowth of multiple dendrites within 5 days.

### Induction of PPIX formation by ALA exposure

PMKs were preincubated for the indicated periods in either low- or high-calcium medium. At given times, ALA was added from the stock solution to yield the final concentration (usually 0.1 mM). At 4 h, 1 ml of the supernatant was aspirated and used for PPIX quantification in the supernatant. The remaining supernatant was removed and the cells were trypsinized. After removal of aliquots for cell number determination (Coulter Counter Model Zf) and protein determination (Bio Rad $D_c$ Protein Assay, Bio Rad Laboratories, Hercules, CA, USA), the remaining cell suspension was dissolved in 3 ml of 1% sodium dodecyl sulphate (SDS) in 0.1 M NaOH. PAM 212, C2C12 and PC12 cells were treated with 0.1 mM ALA with or without the respective, differentiation-inducing pretreatments.

**Figure 1** Time dependence of calcium-induced stimulation of PPIX formation and simultaneous growth arrest. Rates of PPIX accumulation (▲) and [$^{3}$H]Td uptake (□) in PMKs were quantified as a function of time after 2.0 mM calcium addition. 0.1 mM ALA and [$^{3}$H]Td were added 4 h before harvesting the cells. Values are means ± s.d. of triplicate determinations

**Figure 2** ALA concentration dependence of PPIX formation. Accumulation of intracellular PPIX in PMKs after incubation in different ALA concentrations for 4 h with (▲) or without (○) 20 h preincubation in high (2.0 mM) calcium medium

**Figure 3** Calcium concentration dependence of stimulated PPIX accumulation. Intracellular PPIX accumulation (▲) and [$^3$H]Td uptake (□) was determined after preincubation of PMKs in various concentrations of calcium for 20 h and subsequent addition of 0.1 mM ALA and [$^3$H]Td for 4 h

### PPIX quantification

Spectrofluorometry was performed using excitation at 400 nm and recording the emission spectra between 600 and 720 nm on a SPEX-Fluorolog spectrophotometer (SPEX Industries, Edison, NJ, USA). The results were quantified (peak area) using PPIX standards of known concentrations. The recording of the whole spectrum (rather than small-band measurements) allowed for correction for scattering and for exclusion of possible contribution of non-PPIX porphyrins. These can be identified easily in fluorescence spectra obtained in NaOH/SDS solution with an instrument with good resolution as we have shown previously (Iinuma et al, 1994). The supernatant aliquots were diluted with 2 ml of 1% SDS in 1 M NaOH and quantified identically. No porphyrins except PPIX were found in PMKs, PAM 212 and PC12 cells. A shoulder or small peak at 617 nm indicated the presence of small amounts of non-PPIX porphyrins in C2C12 cells. The non-PPIX porphyrins were excluded from quantification.

**Figure 4** Photodynamic sensitization. Cells were preincubated for 20 h in low (○) or high (▲) calcium medium, exposed to 0.1 mM ALA for 4 h and subsequently exposed to monochromatic 630-nm laser light. Surviving fractions are depicted as percentages (means ± s.d. of three samples) of untreated cells. **A**, PMKs. **B**, PAM 212 cells

### Time course experiments

In order to study the time dependence of the calcium-induced increase in intracellular PPIX accumulation, PMKs were exposed to 2.0 mM calcium for up to 24 h. For a final 4-h pulse, ALA and tritiated thymidine were added to reach final concentrations of 0.1 mM and 2 μCi ml$^{-1}$ respectively. After washing twice with PBS, cells were harvested and used for quantification of cell number, protein content, PPIX formation and thymidine uptake.

### Photosensitization experiments

Cells were incubated in low- or high-calcium medium for 20 h. ALA was then added to a final concentration of 0.1 mM and incubation allowed to continue. After 4 h the medium was replaced with 0.5 ml of PBS (after one rinse with PBS). Irradiations were 2.0, 5.0, 7.0, 12.0 and 20.0 J cm$^{-2}$ and delivered at a dose rate of 0.065–0.070 W cm$^{-2}$. An argon-ion pumped dye laser (Coherent, Palo Alto, CA, USA) provided 630-nm light. Fresh complete medium was replaced after the irradiation and the cells returned to the incubator. Twenty-four hours later cells were incubated with

*© Cancer Research Campaign 1998*

**Table 1** Protoporphyrin IX production by cells exposed for 4 h to 0.1 mM ALA. Comparison of intracellular, extracellular and total PPIX quantities in cells grown in low-calcium or after 24 h in high-calcium medium (PMKs, PAM 212). C2C12 myoblasts and PC12 cells were induced to differentiate as described in Material and methods. The ratios show the values of high vs low calcium treated, or differentiated vs undifferentiated cells respectively

| | | PPIX (fmol × $10^{-5}$) production/cell | | | | | |
|---|---|---|---|---|---|---|---|
| **Cells (no. of experiments)** | **[$Ca^{2+}$]** | **Intracellular (s.d.)** | **Ratio (s.d.)** | **Supernatant (s.d.)** | **Ratio (s.d.)** | **Total (s.d.)** | **Ratio (s.d.)** |
| Prim KC (9) | High | 8.42 (2.15) | 7.42 (3.04) | 4.13 (3.23) | 1.55 (1.25) | 12.55 (3.40) | 2.95 (0.98) |
| | Low | 1.24 (0.43) | | 2.98 (1.17) | | 4.22 (1.42) | |
| PAM 212 (3) | High | 2.32 (0.56) | 1.20 (0.37) | 6.93 (2.13) | 0.78 (0.22) | 9.27 (2.60) | 0.80 (0.25) |
| | Low | 2.14 (0.96) | | 9.71 (5.83) | | 11.85 (6.50) | |
| C2C12 (5) | Differentiated | 1.70 (0.42) | 8.63 (3.63) | 4.84 (1.02) | 3.36 (1.74) | 6.54 (1.59) | 3.66 (1.82) |
| | Undifferentiated | 0.23 (0.12) | | 1.62 (0.32) | | 1.85 (0.40) | |

**Figure 5** ALA uptake. Incubation of undifferentiated (○) and differentiated (▲) PMKs in 0.1 mM ALA containing a radiolabelled fraction. ALA dehydratase is blocked by addition of succinyl acetone 15 min before ALA exposure

**Figure 6** Efflux of intracellularly synthesized PPIX. PMKs were preincubated for 20 h in high- or low-calcium medium. Subsequently, ALA was added to a final concentration of 0.1 mM (proliferating cells, ○) or 0.02 mM (differentiated cells, ▲) ALA. The ALA-containing medium was removed after 4 h and replaced by fresh medium without ALA. The cellular PPIX content was determined at the indicated times after ALA removal. Values are means ± s.d. of two determinations

0.5 mg $ml^{-1}$ MTT for 1 h to assay its reduction by mitochondrial dehydrogenases. Mitochondrial dehydrogenase activity provides a sensitive way of assessing cellular damage and has been shown to correlate well with other established measures of cytotoxicity such as colony formation (Iinuma et al, 1994). The latter could not be used in this study as PMKs do not form colonies. Supernatants were carefully removed and the attached cells agitated with 1 ml of DMSO for 30 min on a rotational shaker. The reaction product formazan was quantified photometrically at 576 nm.

### ALA uptake

Cells were grown as described above under 'Photosensitization experiments'. We used succinyl acetone as a potent inhibitor of haem synthesis (Ebert et al, 1979). The cells were incubated in 0.1 mM succinyl acetone for 15 min before 0.1 mM ALA and 0.5 μCi of [$^{14}C$]ALA per dish were added for 5 min, 2 h and 4 h. Following incubation, cells were rinsed 1× with cold medium containing unlabelled ALA, 1× with cold PBS containing unlabelled ALA and trypsinized. Samples were taken for quantification of $^{14}C$ (Beckman Model LS3801 scintillation counter), cell number, protein content and PPIX content (see above).

### Haem enzyme mRNA levels

Total RNA was obtained from 100-mm dishes that were used at the same density and other conditions as the functional experiments. Aliquots of 30 μg were fractionated on a 1.2% denaturing agarose–formaldehyde gel. After transfer to a Hybond N membrane (Amersham) using 20 × SSPE, the filter was dried and the RNA cross-linked to the filter by exposure to 1.2 kJ $m^{-2}$ UVC (Stratalinker, Stratagene). Prehybridization was carried out in 5 × SSPE, 5 × Denhardt's solution, 0.5% SDS and 40 μg $ml^{-1}$ purified salmon sperm DNA (Sigma) at 60°C for 12–20 h. Murine c-DNAs were obtained from Dr H Kohno (coproporphyrinogen oxidase; Kohno et al, 1993) and Dr TR Bishop (ALA dehydratase; Bishop et al, 1989). Membranes were incubated with [$^{32}P$]dCTP random-primed labelled probes (typically > $10^6$ counts $ml^{-1}$) for at least 24 h at 60°C. Two 10-min washes each of 2 × SSPE with 0.1% SDS (25°C), 1 × SSPE with 0.1% SDS (60°C) and 0.5 × SSPE and 0.1% SDS (60°C) preceded autoradiography at –80°C.

### RESULTS

### Keratinocytes undergoing $Ca^{2+}$-induced differentiation show increased accumulation of PPIX

Primary mouse keratinocytes (PMKs) proliferate in the presence of medium containing 0.05 mM calcium ('low calcium'). In this

**Figure 7** Effect of differentiation on steady-state mRNA levels of coproporphyrinogen oxidase. **A**, PMKs in low calcium (lane 1) and 1 h (lane 2), 4 h (lane 3) and 24 h (lane 4) after addition of high calcium. ALA-D mRNA is unaltered when densitometrically compared with GAPDH mRNA (a housekeeping gene transcript used for loading control). **B**, PAM 212 cells in low-calcium medium (lane 1) and 24 h after addition of high calcium (lane 2). **C**, Myoblasts, proliferating (lane 1) and in confluent monolayer (lane 2); confluent monolayers incubated in medium with 5% horse serum for 8 h (lane 3), 24 h (lane 4), 48 h (lane 5) and 72 h (lane 6). **D**, Proliferating PC12 cells (lane 1) and 5 days after addition of 5 ng $ml^{-1}$ (lane 2) or 25 ng $ml^{-1}$ NGF (lane 3). 18S and 28S indicate ribosomal RNA bands presented for RNA loading control system, terminal differentiation and growth arrest can be induced by increasing the calcium concentration in the medium to 2.0 mM, as described previously (Hennings et al, 1980; Hennings and Holbrook, 1983). To determine the effects of growth arrest and terminal differentiation upon ALA-induced PPIX accumulation, we incubated PMKs for various lengths of time in the differentiation-promoting 'high-calcium' medium and harvested the cells after a 4-h pulse of ALA. Identically treated cells were pulsed with tritiated thymidine as shown in Figure 1. Intracellular PPIX accumulation increased with time after inducing differentiation and growth arrest as determined by a decrease in [$^3$H]Td incorporation rate.

Calcium-stimulated PPIX accumulation was demonstrated over a wide ALA concentration range. At a low ALA concentration (0.03 mM) the PPIX concentration was close to the detection limit in proliferating PMKs, but showed almost tenfold higher values in differentiated cells, whereas at the other extreme (0.3 mM ALA), the ratio of intracellular PPIX content between differentiating and undifferentiated cells became smaller than at lower concentrations (Figure 2). Therefore, an intermediate ALA concentration of 0.1 mM was chosen for the majority of experiments.

Previous work has established that markers of keratinocyte differentiation are induced in a calcium concentration-dependent way (Yuspa et al, 1989; Hennings et al, 1980). Therefore, we performed the experiment shown in Figure 3, in which cells were exposed for 24 h to a series of increasing calcium concentrations and induced to differentiate. For the final 4 h of incubation 0.1 mM ALA was added to the medium to stimulate PPIX synthesis. Induction of differentiation by calcium concentrations between 0.25 and 2.0 mM resulted in similar degrees of stimulated PPIX accumulation (Figure 3). Simultaneously, reduced [$^3$H]Td uptake was recorded, showing growth arrest.

### Increased PPIX accumulation leads to greater photosensitization of differentiated vs proliferating keratinocytes

Figure 4A shows fluence-dependent cytotoxicity of ALA-treated proliferating PMKs after 630-nm laser irradiation. The increase in ALA-induced intracellular PPIX in differentiated PMKs resulted in higher photoxicity (Figure 4A). The fluence required for killing 50% of the differentiated PMKs was almost four times lower (2.55 vs 9.80 J $cm^{-2}$) than that for proliferating cells. In this specific experiment, PPIX concentrations were 11-fold higher in differentiating than in proliferating cells (Figure 4A).

The correlation between elevated PPIX concentrations and elevated photosensitization was strengthened by another experiment, in which the keratinocyte cell line PAM 212, which is defective in its ability to differentiate in response to increased calcium concentration in the medium, showed little alteration in ALA-induced PPIX accumulation (Table 1). Photosensitization was likewise almost unaltered in PAM 212 cells in response to high-calcium treatment (Fig. 4B). This inability of different calcium concentrations to affect ALA-induced PPIX formation in PAM cells also indicates that the effect in PMKs is differentiation dependent.

### Several mechanisms contribute to increased PPIX accumulation in differentiating keratinocytes

Three potential factors contributing to the increase in PPIX concentration in differentiating cells were considered and tested experimentally: (a) an increased ALA uptake, (b) a decreased PPIX efflux into the culture medium and (c) an increased synthetic capacity of PPIX.

To assess the effect of differentiation upon ALA uptake, [$^{14}$C]ALA was quantified by measuring intracellular $^{14}$C levels up to 4 h after adding ALA to the medium. To reduce potential confounding effects of (1) differential loss of PPIX into the media from proliferating vs differentiated PMKs (see below), and (2) release of newly synthesized [$^{14}$C]PPIX into the medium, PPIX synthesis was blocked by adding the potent ALA dehydratase inhibitor succinyl acetone before ALA exposure. Figure 5 shows that in differentiated PMKs the ALA uptake was 40% higher than in proliferating cells. Thus, elevated ALA uptake probably contributes to increased PPIX accumulation in differentiated PMKs.

The data in Table 1 address the role of PPIX efflux into the medium. With exposure of proliferating PMKs to 0.1 mM ALA for 4 h, only 29% of the total PPIX was found in the cells. In contrast, differentiating PMKs retained 67% of total PPIX inside the cells (Table 1). That much of the relative difference between differentiating and proliferating keratinocytes in cellular PPIX content could be due to different efflux rates was suggested by experiments specifically designed to measure efflux (Figure 6). Time-dependent loss of ALA-induced PPIX from differentiated and undifferentiated cells was compared in cells that began at the same intracellular PPIX concentrations: undifferentiated PMK lost over 90% of the intracellular PPIX into the medium within 4 h,

© Cancer Research Campaign 1998

whereas, during the same period, differentiated cells lost only 63% (Figure 6).

The differentiation-related decrease in PPIX efflux accounts to some extent for higher intracellular PPIX concentrations of differentiated cells. However, if intracellular and extracellular amounts were combined, it was evident that the total PPIX production was increased in differentiating keratinocytes (Table 1).

### Analysis of haem enzyme transcription

From the above measurements, the increase in the total ALA-induced PPIX levels produced in differentiating PMKs was calculated to be about three times that synthesized by undifferentiated PMKs. In order to understand this altered synthetic capacity, we analysed steady-state mRNA levels of two haem synthetic enzymes. We chose ALA dehydratase, which is the enzyme that synthesizes porphobilinogen from ALA. The other one was coproporphyrinogen oxidase, which forms protoporphyrinogen, the immediate precursor to PPIX. Coproporphyrinogen oxidase mRNA levels increased about fourfold within 24 h of high-calcium exposure. ALA dehydratase mRNA was unaltered over the whole time course (Figure 7A).

### Increased PPIX accumulation is specifically associated with calcium-dependent differentiation

In order to understand the mechanisms resulting in the cellular PPIX increase, we analysed the effect of stimulators of certain aspects of the differentiation response to increased calcium concentration, with respect to modulation of PPIX accumulation. TGF-$\beta_1$ has been shown to induce growth arrest, but does not initiate the differentiation programme in PMKs (Filvaroff et al, 1992). As predicted, TGF-$\beta_1$ did not stimulate PPIX accumulation (Figure 8). TPA also leads to growth arrest but triggers some of the molecular signalling responses that are shared by several calcium-dependent pathways (Dlugosz and Yuspa, 1993; Calautti et al, 1996). TPA did not stimulate PPIX accumulation (Figure 8), indicating that the mechanism of PPIX accumulation occurs by aspects of cellular differentiation not shared by the TPA pathway. The calcium ionophore calcimycin (A23187) has been demonstrated to increase intracellular calcium level in PMKs without affecting differentiation (Filvaroff et al, 1994). Calcimycin also failed to stimulate ALA-induced PPIX formation, suggesting that an increased intracellular calcium concentration alone does not stimulate PPIX synthesis and accumulation (Figure 8). Taken together, these results suggested that the increase in cellular PPIX content in PMKs was specific for high-calcium-induced differentiation.

### PAM 212 cells do not differentiate in vitro and thus fail to show increased ALA-mediated photosensitivity

In order to understand the importance of differentiation for stimulated PPIX levels we analysed transformed epidermal mouse keratinocytes with respect to their differentiating and PPIX-forming capability. PAM 212 is a Balb/c keratinocyte cell line that is tumorigenic. The cells grow in both low-calcium and high-calcium medium; the change from low- to high-calcium medium resulted in a subtle change in morphology, but not growth arrest. Levels of coproporphyrinogen oxidase and ALA dehydratase mRNA values were not influenced by high-calcium treatment (Figure 7B). PPIX production and accumulation was little influenced by high-calcium treatment (Table 1). Under all incubation conditions, PAM 212 cells lost the majority of the porphyrin into the media, similar to proliferating PMKs (Table 1). Accordingly, photosensitization was reduced compared with differentiated PMKs and could not be increased by high-calcium preincubation (Figure 4B).

**Figure 8** PPIX accumulation in PMKs exposed to different stimulators or inhibitors. PMKs were treated for 24 h with 2.0 mM calcium, 2.5 ng ml$^{-1}$ TGF-$\beta_1$, 100 ng ml$^{-1}$ TPA, or 2 μM A23187. For the final 4 h, 0.1 mM ALA was added

### Differentiation leads to increased PPIX formation and coproporphyrinogen oxidase mRNA levels in neuroendocrine and myoblast cell lines

In order to evaluate whether the association between differentiation and an increased haem synthetic capacity is shared by other cells, two additional in vitro models of cellular differentiation were analysed with respect to ALA-induced PPIX formation. We also quantified steady-state mRNA levels for coproporphyrinogen oxidase and ALA dehydratase in these cells.

The murine myoblast cell line C2C12 is a model of mesenchymal differentiation that results in the formation of myotubes and has been characterized at the molecular level (Blau et al, 1985). Basal levels of PPIX production were lower in myoblasts than in the epidermis-derived cells (Table 1). Differentiated myoblasts showed the typical myotube formation in a storiform pattern and exhibited a 3.7-fold increase in total PPIX production and 8.6-fold higher intracellular concentrations (Table 1). The increased total PPIX production was accompanied by an up-regulation of coproporphyrinogen oxidase, but not ALA dehydratase mRNA (Figure 7C).

The cell line PC12, which was derived from a transplantable rat phaeochromocytoma, differentiates in response to treatment with NGF (Greene and Tischler, 1976). The progressive formation of multiple dendrites was the morphological sign of differentiation (Tischler and Greene, 1978). The PPIX levels in undifferentiated PC12 cells treated with 0.1 mM ALA were below detection limit,

© Cancer Research Campaign 1998

but differentiated PC12 cells accumulated 0.55 fmol per cell. We were also able to demonstrate that, after 5 days of NGF induction and after reaching dendritic morphology, coproporphyrinogen oxidase but not ALA dehydratase mRNA levels were increased (Figure 7D).

### DISCUSSION

We have demonstrated that the ability of differentiating PMKs to accumulate PPIX when exposed to exogenous ALA is increased compared with that of undifferentiated, proliferating cells. We also showed that this increase in the cellular PPIX content leads to improved photodynamic sensitization. Lethal phototoxicity was increased by a lesser magnitude than PPIX concentrations. This may indicate altered subcellular distribution and/or enhanced resistance to PDT in differentiated cells. We identified several factors that contributed to the increase in PPIX present in differentiated cells: (a) an increased uptake of ALA, (b) a decreased efflux of PPIX into the culture media and (c) an increase in mRNA for at least one late haem synthetic enzyme, coproporphyrinogen oxidase, a finding consistent with the increased PPIX production.

These results are in contrast to prior reports in which increased ALA-induced PPIX formation was attributed to high rates of proliferation (Iinuma et al, 1994; Rittenhouse-Diakun et al, 1995). This correlation was reported for malignantly transformed cells and for lymphocytes after mitogen stimulation. It has also been demonstrated that the increased expression of transferrin receptor (CD71) was associated with increased PPIX accumulation in both mitogen-stimulated lymphocytes from normal volunteers and in cells obtained from a patient with T cell lymphoma (Rittenhouse-Diakun et al, 1995). However, not all of these studies accounted for PPIX loss into the media, which may cause large differences in cellular levels, as our results have demonstrated.

We analysed the association between the calcium-induced differentiation and the increased cellular PPIX content by studying the effects of exogenous compounds that mimic certain aspects of high-calcium-induced PMK differentiation. The composition of the culture media, such as the FCS concentration, can certainly modify the intracellular PPIX concentration by increasing the export rate (Steinbach et al, 1995). It is feasible that the calcium concentration in the medium may influence the cells' ability to retain PPIX. Such an immediate effect of increased calcium concentration on the ALA-dependent porphyrin accumulation could be excluded because of the gradually increasing magnitude of the PPIX production and accumulation (Figure 1). Also, when differentiated cells were incubated with ALA in low-calcium medium, increased PPIX accumulation prevailed, which was characteristic of differentiated PMKs (data not shown).

The effects of inducers of growth arrest and of a calcium ionophore supported the concept that a full calcium-induced differentiation programme is required for stimulated PPIX accumulation. TPA triggers molecular signals that are also induced by the calcium response; these signals led to partial differentiation and growth arrest (Dlugosz and Yuspa, 1993; Calautti et al, 1996) and did not stimulate but rather suppressed PPIX formation (Figure 8). TGF-$\beta_1$ induces growth arrest only, and not differentiation in PMKs. Calcimycin, a calcium ionophore, increases intracellular free calcium concentration in PMKs without elevation of the extracellular calcium level (Filvaroff et al, 1994). Exposure to either of these two compounds did not lead to increased PPIX accumulation (Figure 8). These data emphasize the complexity of changes induced by calcium and confirm that several factors contribute to the increased PPIX accumulation in PMKs. These findings were supported by the strikingly similar increase in ALA-induced PPIX formation in two other models of in vitro cellular differentiation.

In all three models of differentiation in vitro the increased PPIX production was accompanied by an up-regulation of steady-state mRNA of coproporphyrinogen oxidase. In hepatic and erythroblastic cells, ferrochelatase and porphobilinogen deaminase are considered rate limiting (Bottomley and Muller-Eberhard, 1988). However, enzyme hierarchy is not established for cells with low rates of constitutive haem synthesis such as epidermal keratinocytes so that the observations in this study may not be directly translatable in terms of what is known in cells with an active haem synthesis machinery.

We have demonstrated an association between non-erythroblastic differentiation and increased haem synthetic capacity in keratinocytes, myoblasts and neuronal cells. There is only one prior report on the influence of differentiation induction on ALA-induced porphyrin formation in non-haematopoietic cells (Schoenfeld et al, 1994). In analogy to erythroblastic differentiation in vitro, DMSO was used to stimulate PPIX levels in B16 murine melanoma cells. However, the effect of DMSO was not shown to be differentiation specific in B16 cells (Schoenfeld et al, 1994). In PMKs, identical DMSO treatment had no stimulatory effect on PPIX formation (data not shown).

The increase in ALA-induced PPIX accumulation in differentiating cells may indicate an involvement of the haem synthetic pathway in the regulation of non-haematopoietic differentiation. It has been suggested that coproporphyrinogen oxidase may have a regulatory function in differentiation-specific increase in haem enzymatic activities during erythroblastic differentiation (Conder et al, 1991). Alternatively, this could be related to the association of porphyrins, especially PPIX with the so-called peripheral benzodiazepine receptors on mitochondria, which are up-regulated during erythroblastic differentiation (Wang et al, 1984; Taketani et al, 1994).

In summary, our results suggest that the increase in ALA-induced intracellular PPIX accumulation is calcium specific and differentiation dependent. The close association with differentiation is also emphasized by the striking similarity of increased cellular PPIX levels and increased total PPIX production in PMKs, myoblasts and adrenal cells. In all these cells, the increased PPIX formation was accompanied by increased steady-state mRNA levels of coproporphyrinogen oxidase. The data do not exclude the contribution of other enzymes such as porphobilinogen deaminase or ferrochelatase. Our data do, however, support the importance of differentiation in ALA-induced PPIX formation. Consequently more differentiated tumours or chemotherapy-insensitive tumours with slowly cycling cells may be the better targets of ALA-dependent PDT.

### ACKNOWLEDGEMENTS

This work was supported by a grant from the Department of Defense FEL Research Program (ONR no. N00014-94-I-0927); BO is a recipient of a Career Development Award by the Dermatology Foundation. We thank Dr Terry R Bishop and Dr Hirao Kohno for their gifts of haem enzyme c-DNA and DUSA Pharmaceuticals for the gift of ALA. Special thanks to Dr David Prowse for constant help and advice.

© Cancer Research Campaign 1998

### REFERENCES

Battle AM (1993) Porphyrins, porphyrias, cancer and photodynamic therapy – a model for carcinogenesis. *J Photochem Photobiol B* **20**: 5–22

Bishop TR, Hodes ZI, Frelin LP and Boyer SH (1989) Cloning and sequence of mouse erythroid delta-aminolevulinate dehydratase cDNA. *Nucleic Acids Res* **17**: 1775

Blau HM, Pavlath GK, Hardeman EC, Chiu CP, Silberstein L, Webster SG, Miller SC and Webster C (1985) Plasticity of the differentiated state. *Science* **230** 758–766

Bottomley SS and Muller-Eberhard U (1988) Pathophysiology of heme synthesis. *Semin Hematol* **25**: 282–302

Calautti E, Missero C, Stein PL, Ezzell RM and Dotto GP (1996) fyn tyrosine kinase is involved in keratinocyte differentiation control. *Genes Dev* **9**: 2279–2291

Conder LH, Woodard SI and Dailey HA (1991) Multiple mechanisms for the regulation of haem synthesis during erythroid cell differentiation. Possible role for coproporphyrinogen oxidase. *Biochem J* **275**: 321–326

Dlugosz AA and Yuspa SH (1993) Coordinate changes in gene expression which mark the spinous to granular cell transition in epidermis are regulated by protein kinase C. *J Cell Biol* **120**: 217–255

Ebert PS, Hess RA, Frykholm BC and Tschudy DP (1979) Succinylacetone, a potent inhibitor of heme biosynthesis: effect on cell growth, heme content and delta-aminolevulinic acid dehydratase activity of malignant murine erythroleukemia cells. *Biochem Biophys Res Commun* **88**: 1382–1390

Fijan S, Hönigsmann H and Ortel B (1995) Photodynamic therapy of epithelial skin tumors using delta-aminolaevulinic acid and desferrioxamine. *Br J Dermatol* **133**: 282–288

Filvaroff E, Calautti E, McCormick F and Dotto GP (1992) Specific changes of Ras GTPase-activating protein (GAP) and a GAP-associated p62 protein during calcium-induced keratinocyte differentiation. *Mol Cell Biol* **12**: 5319–5328

Filvaroff E, Calautti E, Reiss M and Dotto GP (1994) Functional evidence for an extracellular calcium receptor mechanism triggering tyrosine kinase activation associated with mouse keratinocyte differentiation. *J Biol Chem* **269**: 21735–21740

Fisher AM, Murphree AL and Gomer CJ (1995) Clinical and preclinical photodynamic therapy. *Lasers Surg Med* **17**: 2–31

Fujita J, Yamamoto M, Yamagami T, Hayashi N, Bishop TR, De Verneuil H, Yoshinaga T, Shibahara S, Morimoto R and Sassa S (1991) Sequential activation of genes for heme pathway enzymes during erythroid differentiation of mouse Friend virus-transformed erythroleukemia cells. *Biochim Biophys Acta* **1090**: 311–316

Grant WE, Hopper C, MacRobert AJ, Speight PM, Bown SG, Loh CS, MacRobert AJ, Bedwell J, Regula J, Krasner N and Bown SG (1993) Photodynamic therapy of oral cancer: photosensitisation with systemic aminolaevulinic acid. *Lancet* **342**: 147–148

Greene LA and Tischler AS (1976) Establishment of a noradrenergic clonal line of rat adrenal pheochromocytoma cells which respond to nerve growth factor. *Proc Natl Acad Sci USA* **73**: 2424–2428

Halling BP, Yuhas DA, Fingar VF and Winkelman JW (1993) Protoporphyrinogen oxidase inhibitors for tumor therapy. In *Porphyric Pesticides: Chemistry, Toxicology, and Pharmaceutical Applications*, ACS Symposium Series No. 559, Duke, SO and Rebeiz, CA (eds), pp. 280–290. American Chemical Society: Chicago, IL.

Hasan T and Parrish JA (1996) Photodynamic therapy of cancer. In *Cancer Medicine*, Holland JF, Frei E, Bast RC, Kufe DW, Morton DL and Weichselbaum RR (eds), pp. 739–751. Williams & Wilkins: Baltimore.

Henderson BW and Dougherty TJ (1992) How does photodynamic therapy work? *Photochem Photobiol* **55**: 145–157

Henderson BW, Vaughan L, Bellnier DA, van Leengoed H, Johnson PG and Oseroff AR (1995) Photosensitization of murine tumor, vasculature and skin by 5-aminolevulinic acid-induced porphyrin. *Photochem Photobiol* **62**: 780–789

Hennings H and Holbrook (1983) Calcium regulation of cell–cell contact and differentiation of epidermal cells in culture: an ultrastructural study. *Exp Cell Res* **143**: 127–142

Hennings H, Michael D, Cheng C, Steinert P, Holbrook K and Yuspa SH (1980) Calcium regulation of growth and differentiation of mouse epidermal cells in culture. *Cell* **19**: 245–254

Hua Z, Gibson SL, Foster TH and Hilf R (1995) Effectiveness of delta-aminolevulinic acid-induced protoporphyrin as a photosensitizer for photodynamic therapy in vivo. *Cancer Res* **55**: 1723–1731

Iinuma S, Farshi SS, Ortel B and Hasan T (1994) A mechanistic study of cellular photodestruction with 5-aminolaevulinic acid-induced porphyrin. *Br J Cancer* **70**: 21–28

Kennedy JC and Pottier RH (1992) Endogenous protoporphyrin IX, a clinically useful photosensitizer for photodynamic therapy. *J Photochem Photobiol B* **14**: 275–292

Kohno H, Furukawa T, Yoshinaga T, Tokunaga R and Taketani S (1993) Coproporphyrinogen oxidase. Purification, molecular cloning, and induction of mRNA during erythroid differentiation. *J Biol Chem* **268**: 21359–21363

Loh CS, MacRobert AJ, Bedwell J, Regula J, Krasner N and Bown SG (1993) Oral versus intravenous administration of 5-aminolaevulinic acid for photodynamic therapy. *Br J Cancer* **68**: 41–51

Malik Z, Kostenich G, Roitman L, Ehrenberg B and Orenstein A (1995) Topical application of 5-aminolevulinic acid, DMSO and EDTA: protoporphyrin IX accumulation in skin and tumours of mice. *J Photochem Photobiol B* **28**: 213–218

Ortel B, Calzavara-Pinton PG, Szeimies RM and Hasan T (1996) Perspectives in cutaneous photodynamic sensitization. *J Photochem Photobiol B* **36**: 209–211

Ortel B, Tanew A and Hönigsmann H (1993) Lethal photosensitization by endogenous porphyrins of PAM cells – modification by desferrioxamine. *J Photochem Photobiol B* **17**: 273–278

Regula J, Ravi B, Bedwell J, MacRobert AJ and Bown SG (1994) Photodynamic therapy using 5-aminolaevulinic acid for experimental pancreatic cancer – prolonged animal survival. *Br J Cancer* **70**: 248–254

Riesenberg R, Fuchs C and Kriegmair M (1996) Photodynamic effects of 5-aminolevulinic acid-induced porphyrin on human bladder carcinoma cells in vitro. *Eur J Cancer* **32A**: 328–334

Rimington C (1989) Haem biosynthesis and porphyrias: 50 years in retrospect. *J Clin Chem Clin Biochem* **27**: 473–486

Rittenhouse-Diakun K, Van Leengoed H, Morgan J, Hryhorenko E, Paszkiewicz G, Whitaker JE and Oseroff AR (1995) The role of transferrin receptor (CD71) in photodynamic therapy of activated and malignant lymphocytes using the heme precursor delta-aminolevulinic acid (ALA). *Photochem Photobiol* **61**: 523–528

Sassa S (1976) Sequential induction of heme pathway enzymes during erythroid differentiation of mouse Friend leukemia virus-infected cells. *J Exp Med* **143**: 305–315

Schoenfeld N, Mamet R, Nordenberg Y, Shafran M, Babushkin T and Malik Z (1994) Protoporphyrin biosynthesis in melanoma B16 cells stimulated by 5-aminolevulinic acid and chemical inducers: characterization of photodynamic inactivation. *Int J Cancer* **56**: 106–112

Steinbach P, Weingandt H, Baumgartner R, Kriegmair M, Hofstadter F and Knuchel R (1995) Cellular fluorescence of the endogenous photosensitizer protoporphyrin IX following exposure to 5-aminolevulinic acid. *Photochem Photobiol* **62**: 887–895

Svanberg K, Andersson T, Killander D, Wang I, Stenram U, Andersson-Engels S, Berg R, Johansson J and Svanberg S (1994) Photodynamic therapy of non-melanoma malignant tumours of the skin using topical delta-amino levulinic acid sensitization and laser irradiation. *Br J Dermatol* **130**: 743–751

Taketani S, Kohno H, Okuda M, Furukawa T and Tokunaga R (1994) Induction of peripheral-type benzodiazepin receptors during differentiation of mouse erythroleukemia cells. *J Biol Chem* **269**: 7527–7531

Taketani S, Yoshinaga T, Furukawa T, Kohno H, Tokunaga R, Nishimura K and Inokuchi H (1995) Induction of terminal enzymes for heme biosynthesis during differentiation of mouse erythroleukemia cells. *Eur J Biochem* **230**: 760–765

Tischler AS and Greene LA (1978) Morphologic and cytochemical properties of a clonal line of rat adrenal pheochromocytoma cells which respond to nerve growth factor. *Lab Invest* **39**: 77–89

Van Hillegersberg R, Van den Berg JW, Kort WJ, Terpstra OT and Wilson JH (1992) Selective accumulation of endogenously produced porphyrins in a liver metastasis model in rats. *Gastroenterology* **103**: 647–651

Wang JKT, Morgan JI and Spector S (1984) Differentiation of Friend erythroleukemia cells induced by benzodiazepines. *Proc Natl Acad Sci USA* **81**: 3770–3772

Wolf P, Rieger E and Kerl H (1993) Topical photodynamic therapy with endogenous porphyrins after application of 5-aminolevulinic acid. An alternative treatment modality for solar keratoses, superficial squamous cell carcinomas, and basal cell carcinomas? *J Am Acad Dermatol* **28**: 17–21

Yaffe D and Saxel O (1977) A myogenic cell line with altered serum requirements for differentiation. *Differentiation* **7**: 159–166

Yuspa SH, Hawley-Nelson P, Koehler B and Stanley JR (1980) A survey of transformation markers in differentiating epidermal cell lines in culture. *Cancer Res* **40**: 4694–4703

Yuspa SH, Kilkenny AE, Steinert PM and Roop DR (1989) Expression of murine epidermal differentiation markers is tightly regulated by restricted extracellular calcium concentrations in vitro. *J Cell Biol* **109**: 1207–1217

© Cancer Research Campaign 1998